# Shiga toxin production and translocation during microaerobic human colonic infection with Shiga toxin-producing *E. coli* O157:H7 and O104:H4

**DOI:** 10.1111/cmi.12281

**Published:** 2014-03-21

**Authors:** Seav-Ly Tran, Lucile Billoud, Steven B Lewis, Alan D Phillips, Stephanie Schüller

**Affiliations:** 1 Norwich Medical School, University of East AngliaNorwich Research Park, Norwich, UK; 2 Gut Health and Food Safety Programme, Institute of Food ResearchNorwich Research Park, Norwich, UK; 3 Faculty of Science, Aix-Marseille UniversityMarseille, France; 4 Centre for Paediatric Gastroenterology, Royal Free HospitalLondon, UK

## Abstract

Haemolytic uraemic syndrome caused by Shiga toxin-producing *E. coli* (STEC) is dependent on release of Shiga toxins (Stxs) during intestinal infection and subsequent absorption into the bloodstream. An understanding of Stx-related events in the human gut is limited due to lack of suitable experimental models. In this study, we have used a vertical diffusion chamber system with polarized human colon carcinoma cells to simulate the microaerobic (MA) environment in the human intestine and investigate its influence on Stx release and translocation during STEC O157:H7 and O104:H4 infection. Stx2 was the major toxin type released during infection. Whereas microaerobiosis significantly reduced bacterial growth as well as Stx production and release into the medium, Stx translocation across the epithelial monolayer was enhanced under MA versus aerobic conditions. Increased Stx transport was dependent on STEC infection and occurred via a transcellular pathway other than macropinocytosis. While MA conditions had a similar general effect on Stx release and absorption during infection with STEC O157:H7 and O104:H4, both serotypes showed considerable differences in colonization, Stx production, and Stx translocation which suggest alternative virulence strategies. Taken together, our study suggests that the MA environment in the human colon may modulate Stx-related events and enhance Stx absorption during STEC infection.

## Introduction

Shiga toxin-producing *E. coli* (STEC) are the fourth most common bacterial foodborne pathogen and responsible for around 1200 illnesses per year in the UK (Money *et al*., 2010). While most infections resolve spontaneously after a period of acute gastroenteritis and/or haemorrhagic colitis, up to 15% of cases develop haemolytic uraemic syndrome (HUS), a severe systemic complication that can be fatal. HUS is the leading cause of acute kidney failure in children in the western world (Tarr *et al*., 2005). Although STEC have been linked to HUS since 1983, there is still no specific treatment available, and use of antibiotics remains controversial (Karmali *et al*., 1983; Riley *et al*., 1983; Tarr *et al*., 2005; Wong *et al*., 2012).

After ingestion via contaminated food or water, STEC adhere to human intestinal epithelium and inject bacterial effector proteins into the host cell through a type III secretion system (T3SS). These effector proteins interfere with host cell signalling and function (e.g. tight junction integrity and alteration of ion channels) which ultimately leads to loss of absorptive function and diarrhoea (Viswanathan *et al*., 2009; Wong *et al*., 2011). In addition, STEC produce Shiga toxins (Stxs) which are strongly associated with HUS and can be divided into Stx1 and Stx2 and their subtypes (Karmali *et al*., 1985; Bergan *et al*., 2012). Shiga toxins are encoded by lambdoid prophages on the bacterial chromosome and are not secreted via the T3SS but released during bacterial lysis after induction of the phage lytic cycle (Wagner *et al*., 2001b; 2002[Bibr b63],[Bibr b64]). In the host, Stxs bind to a membrane glycolipid receptor (globotriaosylceramie, Gb3) which is highly expressed by microvascular endothelium in the kidney, the primary target for Stxs in human disease (Hughes *et al*., 2002). After endocytosis, Stxs undergo retrograde transport via the Golgi and endoplasmic reticulum before release into the cytoplasm where they cause cell death by inhibition of protein synthesis and subsequent apoptosis (Lingwood *et al*., 1987; Endo *et al*., 1988; Tesh, 2012). Most commonly used human colon carcinoma cell lines express Gb3 probably as a consequence of malignant transformation as Gb3 expression has been linked to cancer (Kovbasnjuk *et al*., 2005; Engedal *et al*., 2011). In contrast, normal human intestinal epithelium appears to lack Gb3 and other Stx binding sites (Björk *et al*., 1987; Holgersson *et al*., 1991; Schüller *et al*., 2004) although this has been debated in a recent study (Zumbrun *et al*., 2010). It remains unknown how Stxs are absorbed into the systemic circulation (Schüller, 2011). Several routes of access such as paracellular leakage during neutrophil transmigration and STEC translocation via M cells have been investigated (Hurley *et al*., 2001; Etienne-Mesmin *et al*., 2011), but Gb3-independent transcytosis by intestinal epithelial cells represents the most studied mechanism so far (Acheson *et al*., 1996; Philpott *et al*., 1997; Malyukova *et al*., 2009). While all transcytosis studies on polarized Gb3-negative human T84 colon carcinoma cells have been performed with purified Stx1, we have studied Stx production, release and translocation during infection with Stx2-producing STEC O157:H7 and O104:H4 bacterial strains. We have focused on Stx2 as this toxin type is more closely associated with HUS than Stx1 (Heuvelink *et al*., 1995; Boerlin *et al*., 1999). In addition, we have employed a microaerobic vertical diffusion chamber (VDC) system (Schüller and Phillips, 2010) to mimic the low oxygen environment in the human gut and study its effect on Stx release and absorption during STEC infection.

## Results

### STEC infection of polarized T84 cells in a microaerobic vertical diffusion chamber

**Figure 1:**
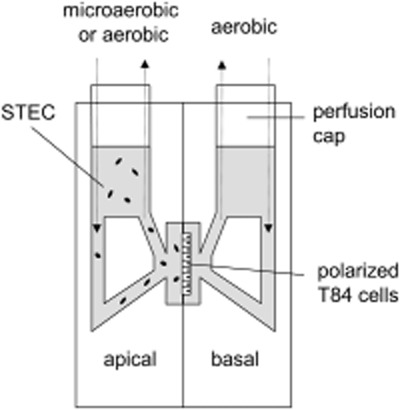
Schematic representation of vertical diffusion chamber. Polarized T84 cells grown on Snapwell filters were inserted between two half chambers and infected apically with STEC. Apical chambers were maintained under aerobic or microaerobic conditions whereas basal compartments were kept under aerobic conditions (modified from Schüller and Phillips, 2010).

**Figure 2:**
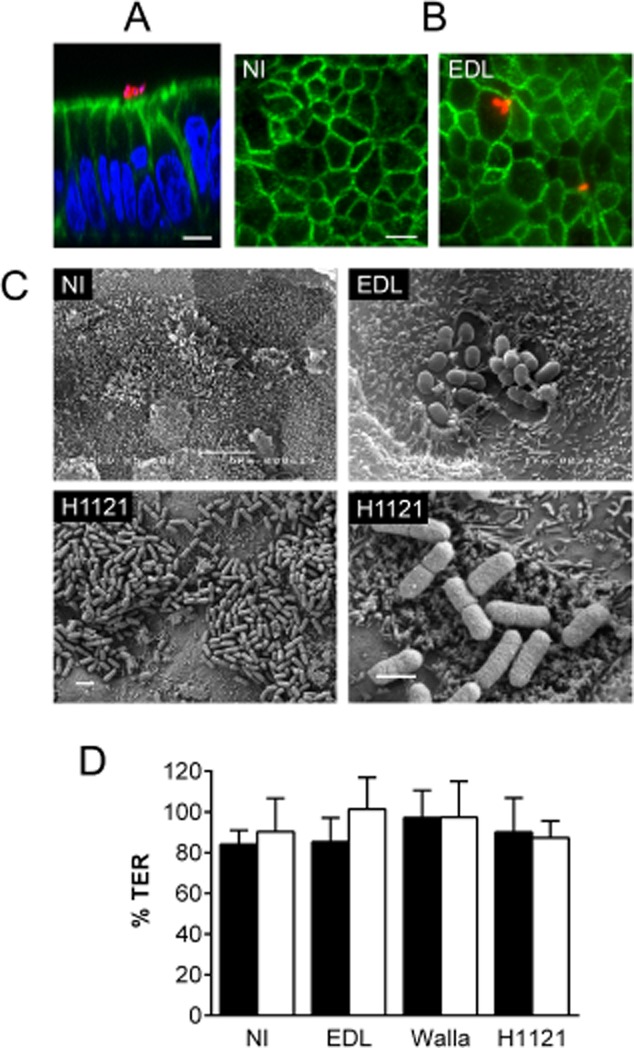
T84 cell integrity and STEC adherence after 5 h infection. Shown are representative images from two independent experiments performed in duplicate (A–C). A. Confocal XZ scan of T84 cells infected with EDL933. Cell monolayers were stained for *E. coli* (red), F-actin (green) and cell nuclei (blue). Bar = 5 μm. B. Immunofluorescence staining of non-infected (NI) and EDL933-infected T84 cells. Cells were stained for occludin (green) and *E. coli* (red). Bar = 10 μm. C. Scanning electron micrographs of non-infected T84 cells (NI) and cells infected with EDL933 or H1121. Bars = 5 μm (NI), 2 μm (H1121, left panel) and 1 μm (EDL933; H1121, right panel). D. TER of non-infected T84 cells (NI) and cells infected with EDL933, Walla-1 or H1121 under AE (■) or MA (□) conditions. TER after infection is expressed as percentage of TER before infection. Data are shown as means ± SEM from seven independent experiments performed in duplicate.

Polarized T84 colon carcinoma cells were mounted in a VDC and infected apically with STEC O157:H7 strains EDL933, Walla-1, or an isolate of the 2011 STEC O104:H4 outbreak in Germany (H1121). Infections were performed for 5 h under either microaerobic (MA, oxygen concentration of 1.4–1.7% atmospheric pressure) or aerobic (AE, 18–20%) conditions on the apical side while the basal side was kept under aerobic conditions (Fig. 1). T84 cell integrity and bacterial adherence were investigated by microscopy. XZ scanning confocal microscopy showed an intact monolayer of columnar shaped T84 cells with an apical actin-rich brush border indicating cell polarization (Fig. 2A). Tight junction integrity and intact barrier function during infection were confirmed by occludin staining (Fig. 2B) and maintenance of stable transepithelial electrical resistance (TER) (Fig. 2D). Scanning electron microscopy (SEM) revealed a dense microvillous brush border particularly on non-infected T84 cells (Fig. 2C). Small bacterial microcolonies were observed on T84 monolayers infected with EDL933 (Fig. 2C) or Walla-1 (data not shown) with evident microvillous effacement around adherent bacteria. In contrast, O104:H4 strain H1121 exhibited extensive adherence of bacterial aggregates. Microvilli around adherent bacteria showed signs of vesiculation but no effacement (Fig. 2C). T84 cell integrity and bacterial adherence patterns were comparable under MA and AE conditions.

### STEC EDL933 growth and Stx release are reduced under microaerobic conditions

**Figure 3:**
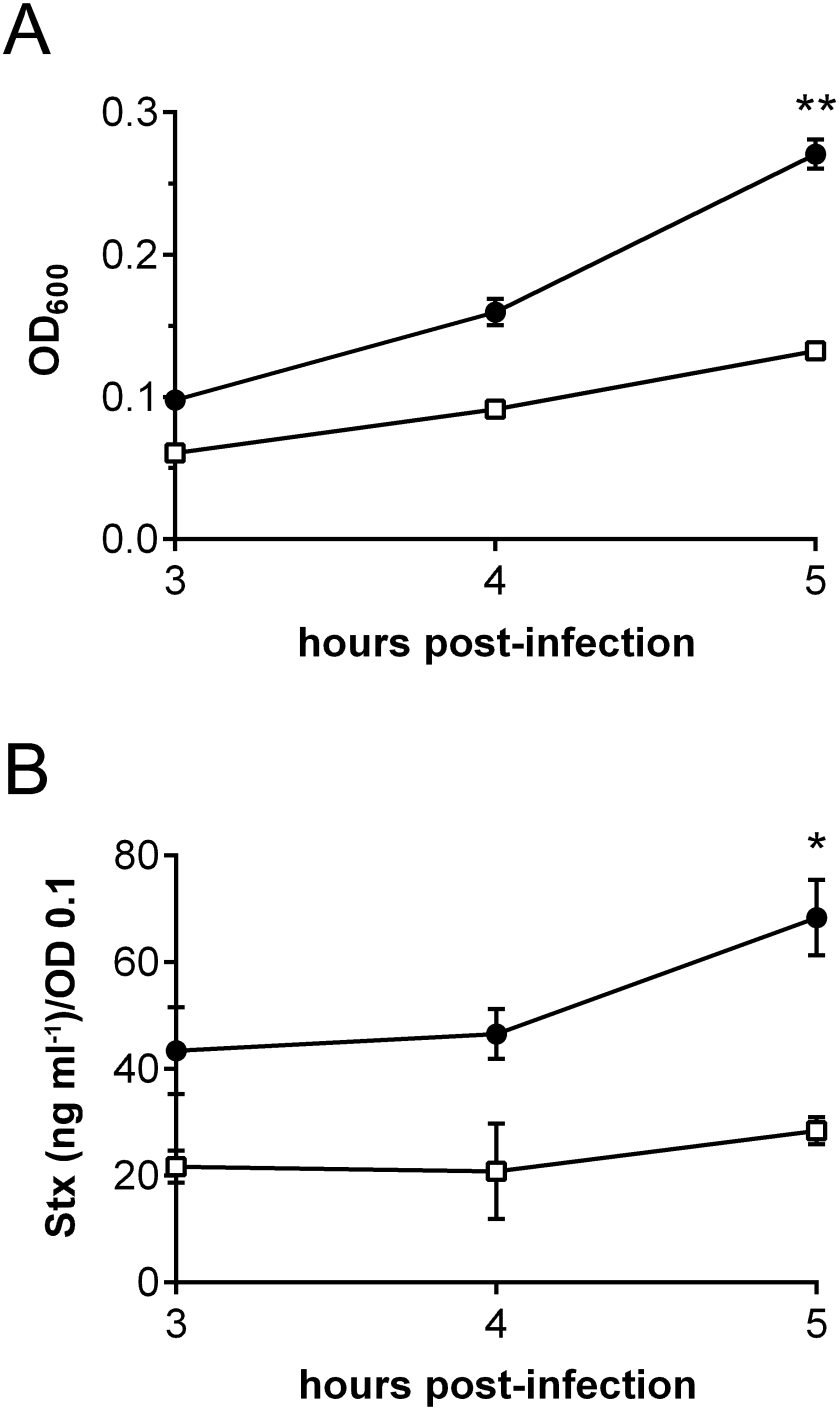
Microaerobic conditions reduce STEC EDL933 growth and Stx release into the apical medium. T84 cells were infected for 3 to 5 h and maintained under AE (●) or MA (□) conditions. Bacterial growth was assessed by OD_600_ (A). Stx cytotoxicity in apical supernatants was determined by VCCA (B). Data are shown as means ± SEM from three independent experiments performed in duplicate. *P < 0.05, **P < 0.01.

To examine the effect of a MA environment on bacterial growth and Stx release into the medium, polarized T84 cells were infected with EDL933 under MA or AE conditions for 3–5 h. Bacterial growth in the apical chamber was assessed by optical density (OD_600_) while Stx release was quantified by Vero cell cytotoxicity assay (VCCA). Stx cytotoxicity was expressed as equivalent concentration of purified Stx2 which was used for the standard curve. Values were adjusted to an OD_600_ of 0.1 to account for differences in bacterial density. As shown in Fig. 3A and B, growth of EDL933 and Stx cytotoxicity in the medium were significantly reduced under MA versus AE conditions after 5 h of infection.

### Stx2 is the major toxin type released during EDL933 infection

**Figure 4:**
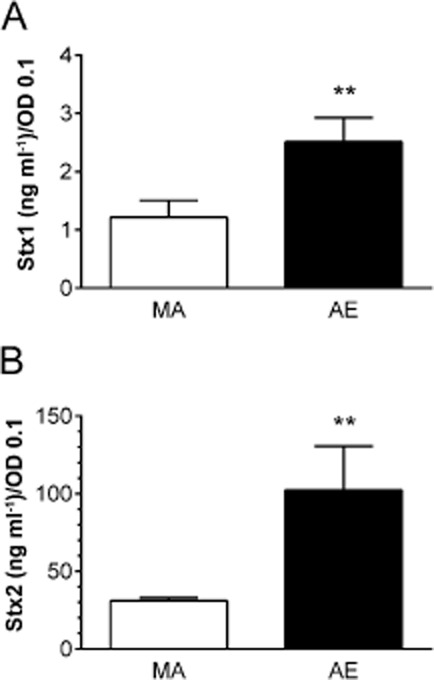
Microaerobiosis decreases Stx release and Stx2 is the main toxin type in supernatants during EDL933 infection. T84 cells were infected for 5 h under AE or MA conditions. Stx1 (A) and Stx2 levels (B) in apical supernatants were quantified by Stx type-specific ELISAs. Data are shown as means ± SEM from five independent experiments performed in duplicate. **P < 0.01.

While the VCCA represents the most sensitive assay to determine Stx levels according to toxin activity (cytotoxicity on Vero cells) it cannot distinguish between Stx1 and Stx2. This is important as EDL933 produces both toxin types. In addition, Stx activity and actual protein levels might not be directly correlated, and decreased Stx cytotoxicity in MA supernatants might be due to loss of activity rather than diminished toxin release. To quantify Stx protein levels in apical supernatants, Stx1- and Stx2-specific sandwich ELISAs were developed and applied. As before, Stx concentrations in supernatants were adjusted to OD 0.1 to account for different bacterial densities in apical media. As shown in Fig. 4A and B, both Stx1 and Stx2 release were significantly reduced under MA versus AE conditions. In addition, Stx2 was the predominant toxin type released into the medium under both AE and MA conditions (98% and 96% of total Stx respectively). This was corroborated by VCCA neutralization assays where addition of anti-Stx2 abrogated more than 95% of cytotoxicity from apical supernatants whereas addition of anti-Stx1 did not show any detectable effect (Fig. S1).

### Stx2 stability and binding to T84 cells are not affected by oxygen levels

**Figure 5:**
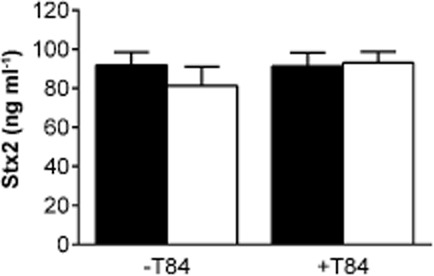
Stx2 stability and T84 cell binding are not affected by oxygen levels. Purified Stx2 (100 ng ml^−1^) was incubated for 5 h with (+ T84) or without polarized T84 cells (− T84) under AE (■) or MA (□) conditions. Stx2 levels were quantified by ELISA. Data are shown as means ± SEM from four independent experiments performed in duplicate.

Lower Stx2 concentrations in the apical medium after MA versus AE EDL933 infection could be caused by reduced Stx release, lower toxin stability or increased Stx binding to T84 cells under MA conditions. To investigate the latter two possibilities, purified Stx2 at a concentration comparable to that released during infection (100 ng ml^−1^) was incubated with or without polarized T84 cells for 5 h under AE or MA conditions. Stx2 levels in the apical medium were quantified by ELISA. As shown in Fig. 5, comparable amounts of Stx2 were recovered under AE and MA conditions without T84 cells indicating that oxygen levels do not influence Stx2 stability. In addition, Stx2 concentrations in apical media incubated with or without T84 cells showed no significant difference, thereby indicating negligible Stx2 binding to polarized T84 cells under AE or MA conditions (Fig. 5).

### Microaerobiosis reduces bacterial growth and Stx2 release in other STEC strains and decreases Stx1 production in EDL933 lysates

**Figure 6:**
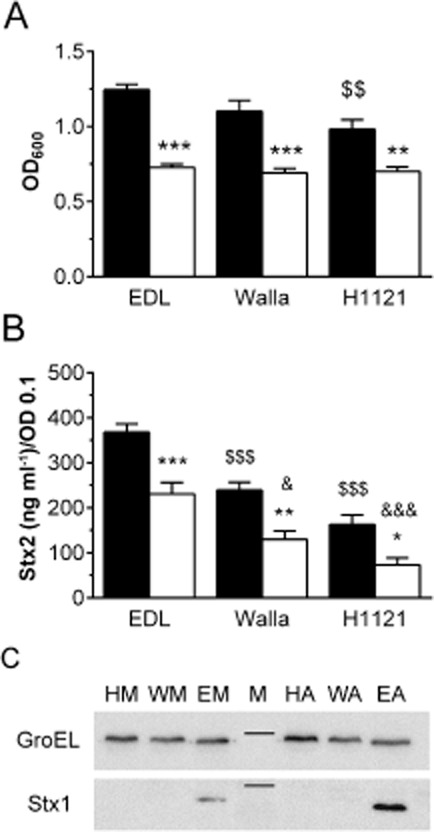
Microaerobiosis reduces growth, Stx production and release in STEC EDL933, Walla-1 and H1121. Polarized T84 cells were infected for 5 h under AE (■) or MA (□) conditions. A. Bacterial growth was assessed by OD_600_. Data are shown as means ± SEM from five independent experiments performed in duplicate. **P < 0.01, ***P < 0.001 versus AE conditions; ^$$^*P* < 0.01 versus EDL933 AE. B. Stx2 levels in apical supernatants were quantified by ELISA. Data are shown as means ± SEM from four independent experiments performed in duplicate. *P < 0.05, **P < 0.01, ***P < 0.001 versus AE; ^$$$^*P* < 0.001 versus EDL933 AE; ^&^*P* < 0.05, ^&&&^*P* < 0.001 versus EDL933 MA. C. Stx1 production in bacterial lysates of EDL933 (E), Walla-1 (W) and H1121(H) under AE (A) and MA (M) conditions was determined by Western blotting. Amounts of loaded bacterial protein were assessed by GroEL expression. The marker lane (M) indicates 58 kDa for GroEL and 30 kDa for Stx1. Shown are representative images from three independent experiments.

Experiments to study the effect of oxygen on bacterial growth and Stx2 release were extended to O157:H7 strain Walla-1 and O104:H4 strain H1121. Both strains produce the same Stx2 subtype as EDL933 (Stx2a) but do not produce Stx1. Similar to EDL933, both strains showed significantly reduced growth and Stx2 supernatant levels under MA versus AE conditions (Fig. 6A and B). In addition, aerobic growth of H1121 was significantly lower compared with EDL933 (Fig. 6A) and Stx2 release by Walla-1 and H1121 was significantly reduced compared with EDL933 under AE and MA conditions (Fig. 6B).

To investigate whether lower Stx release under MA conditions was associated with diminished Stx production within bacteria, Stx expression in bacterial lysates was examined by Western blot analysis. Stx2 levels in lysates of all three STEC strains were equally low under AE or MA conditions and could only be detected after prolonged exposure times (data not shown). In contrast, Stx1 could readily be detected in EDL933 lysates and expression was reduced under MA versus AE conditions (Fig. 6C). As expected, no Stx1 signal was present in lysates of Walla-1 and H1121.

### Stx2 translocation across polarized T84 monolayers is enhanced by microaerobic STEC infection

**Figure 7:**
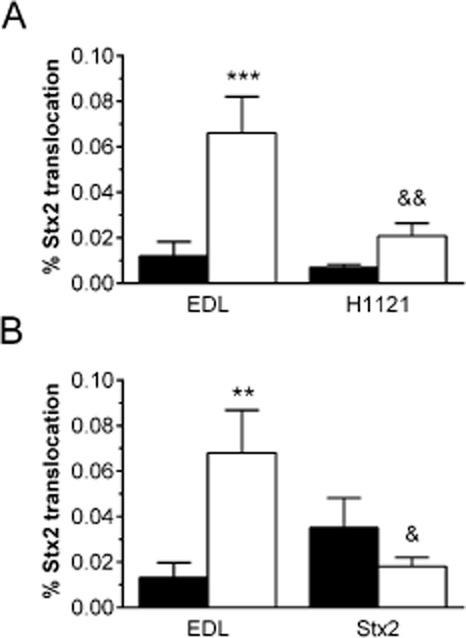
Stx2 translocation across polarized T84 cells is enhanced by STEC infection under MA conditions. T84 cells were apically incubated with EDL933 (A, B), H1121 (A) or purified Stx2 (B) for 5 h under AE (■) or MA (□) conditions. Stx2 concentrations in apical and basal media were determined by VCCA. Translocation rates are expressed as percentage of apical Stx recovered in basal compartments. A. Data are shown as means ± SEM from seven independent experiments performed in duplicate. ***P < 0.001 EDL933 MA versus AE; ^&&^*P* < 0.01 H1121 MA versus EDL933 MA. B. Data are shown as means ± SEM from five independent experiments performed in duplicate. **P < 0.01 versus AE; ^&^*P* < 0.05 versus EDL933 MA.

To determine whether microaerobiosis affects Stx2 translocation across intestinal epithelium, polarized T84 cell monolayers were infected with EDL933 or H1121 and VDC incubations were carried out under MA or AE conditions. After 5 h, media from apical and basal compartments were sampled and Stx concentrations were determined. Initial experiments showed that Stx2 levels in concentrated basal supernatants were too low to detect by ELISA. Therefore, the more sensitive VCCA was used for Stx2 quantification in apical and basal media. Quantification of apical Stx2 levels after EDL933 and H1121 infection by ELISA and VCCA yielded comparable results (Fig. S2). As shown in Fig. 7A, Stx2 translocation was significantly enhanced under MA conditions in EDL933-infected cells. A similar trend was observed in cells infected with H1121 although this did not reach significance. In addition, Stx2 translocation was significantly higher after MA infection with EDL933 when compared with H1121 (Fig. 7A).

To investigate whether enhanced Stx2 translocation under microaerobiosis could be attributed to physiological changes in the host cells, subsequent incubations were performed with either EDL933 or purified Stx2. As shown in Fig. 7B, there was no significant difference between translocation of purified Stx2 under AE versus MA conditions. In addition, Stx2 translocation during MA EDL933 infection was significantly increased compared with purified Stx2 alone (Fig. 7B) indicating a role of the bacterial infection process in Stx absorption.

### Enhanced Stx2 translocation during microaerobic STEC infection is independent of macropinocytosis

**Figure 8:**
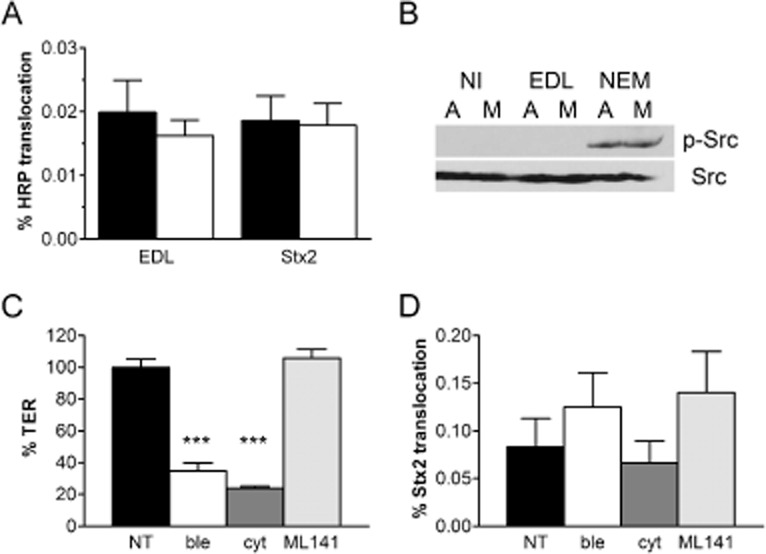
Macropinocytosis is not involved in enhanced Stx2 translocation during microaerobic STEC infection. A. HRP was added to T84 cells incubated with EDL933 or purified Stx2 for 5 h under AE (■) or MA (□) conditions. HRP concentrations in apical and basal media were determined by HRP activity assay. Translocation rates are expressed as percentage of apical HRP recovered in basal compartments. Data are shown as means ± SEM from four independent experiments performed in duplicate. B. Polarized T84 cells were incubated with EDL933, NEM or left non-infected (NI) for 8 h under aerobic (A) or microaerobic conditions (M). After separation of cell lysates by SDS-PAGE, phosphorylated (p-Src) and total Src kinase (Src) were detected by Western blotting. Shown are representative images from two independent experiments performed in duplicate. C and D. Polarized T84 cells were incubated with EDL933 for 5 h under MA conditions. Apical sides were either left untreated (NT) or incubated with cytochalasin D (cyt), blebbistatin (ble) or ML141. TER (C) and Stx2 translocation (D) was determined after infection. Data are shown as means ± SEM from four independent experiments performed in triplicate. ***P < 0.001 versus NT or ML 141.

Recent studies have demonstrated STEC-induced Stx uptake by macropinocytosis (Lukyanenko *et al*., 2011; In *et al*., 2013). To determine whether this pathway was also involved in Stx translocation in our experimental system, we investigated several indicators of macropinocytosis. During morphological examination of STEC-infected polarized T84 cells described earlier, we did not observe any signs of membrane ruffling, blebbing or actin reorganization (Fig. 2A and C). In another set of experiments, polarized T84 cells were incubated with EDL933 or purified Stx2 under AE or MA conditions and the fluid-phase marker horseradish peroxidase (HRP) was also added to the apical medium. As shown in Fig. 8A, translocation of HRP was not significantly affected by MA infection.

We further investigated the involvement of Src kinase activation which had been linked to Stx1 macropinocytosis in earlier studies (Malyukova *et al*., 2009). Infections with EDL933 under MA or AE conditions were performed for 4 to 8 h, and T84 cell lysates were examined for Src kinase phosphorylation by Western blot analysis. As a positive control, T84 cells were incubated with *N*-ethylmaleimide (NEM) for 4 h to induce Src phosphorylation. As shown in Fig. 8B, neither microaerobiosis nor EDL933 infection triggered Src activation after 8 h of incubation. Similar results were obtained after shorter incubations of 4 and 6 h (data not shown).

Finally, we tested several inhibitors of macropinocytosis which had previously been shown to decrease Stx1 uptake and/or translocation (Lukyanenko *et al*., 2011; In *et al*., 2013). These included cytochalasin D (inhibitor of actin polymerization), blebbistatin (inhibitor of non-muscle myosin II ATPase) and ML 141 (inhibitor of Cdc42 GTPase). Polarized T84 cells were infected with EDL933 for 5 h under MA conditions, and inhibitors were added to the apical chambers. None of the reagents affected bacterial growth or Stx release into the medium (data not shown). However, a significant loss of TER was observed in cytochalasin D and blebbistatin-treated T84 monolayers (Fig. 8C). This was not observed with DMSO which was used as a solvent for all reagents (data not shown). As shown in Fig. 8D, none of the inhibitors significantly affected Stx2 translocation across the T84 monolayer.

## Discussion

Oxygen levels in the human intestine are considered anaerobic in the lumen and microaerobic at the mucosal surface due to oxygen diffusion from the epithelium (Marteyn *et al*., 2011). Although it is difficult to determine accurate oxygen levels in the human gut, data obtained by non-invasive imaging in the mouse indicate 1.4% oxygen in the mid-small intestine and colon (He *et al*., 1999). Therefore, it is important to consider the influence of reduced oxygen levels on virulence of intestinal pathogens, and several recent studies have demonstrated virulence gene modulation in response to oxygen (Marteyn *et al*., 2011). The vertical diffusion chamber system presented in this study has been successfully used to demonstrate enhanced adherence, invasion and host cell response to infection with *Helicobacter pylori* and *Campylobacter jejuni* under microaerobiosis (Cottet *et al*., 2002; Mills *et al*., 2012). In addition, previous studies have shown that low oxygen concentrations increase STEC adherence to small and large intestinal epithelial cells by promoting T3S and expression of Sfp fimbriae (Müsken *et al*., 2008; Schüller and Phillips, 2010). In this study, we have focused on the aspect of Stx interaction with human colonic epithelium, as STEC pathology is most predominant in the colon (Tarr *et al*., 2005). We have used polarized T84 human colon carcinoma cells which like human intestinal epithelium do not express the Stx receptor Gb3 and are resistant to Stx cytotoxicity (Schüller *et al*., 2004). Similar to our previous study with Stx-negative STEC (TUV 93-0) (Schüller and Phillips, 2010), infection with wild-type STEC did not cause disruption of barrier function after 5 h of infection, thereby excluding paracellular leakage of Stxs through the cell monolayer (Philpott *et al*., 1997; Hurley *et al*., 2001). Adherence patterns of O157:H7 strains EDL933 and Walla-1 were comparable to those observed with TUV 93-0 demonstrating small bacterial microcolonies with localized microvillous effacement (Schüller and Phillips, 2010). In contrast, colonization by the O104:H4 isolate H1121 was far more extensive and revealed formation of bacterial aggregates and surrounding microvillous vesiculation characteristic for enteroaggregative *E. coli* (EAEC) (Hicks *et al*., 1996; Nataro *et al*., 1996)*.* A similar adherence phenotype has also been described by Bielaszewska *et al*. (2011) and is in agreement with the EAEC genetic backbone of O104:H4 (Rasko *et al*., 2011).

When we investigated bacterial growth under AE and MA conditions, growth of all three STEC strains was reduced under microaerobiosis. *E. coli* bacteria express two respiratory oxidases for oxygen reduction. The low-affinity cytochrome *bo_3_* oxidase is used at high oxygen levels, whereas the high-affinity cytochrome *bd* oxidase is active at low oxygen concentrations but is energetically less efficient (Gunsalus and Park, 1994; Jones *et al*., 2007).

After ingestion via contaminated food or water, STEC colonize the human intestine and release Stxs. Stxs are encoded by the late genes of lambdoid prophages and Stx2 expression and release is mainly regulated by the late phage promoter *p*_R_’ and therefore dependent on the phage lytic cycle (Wagner *et al*., 2001b). Spontaneous prophage induction occurs at very low frequency but a recent study in germ-free mice has shown that the intestinal environment triggers the Stx2 phage lytic cycle (Tyler *et al*., 2013). *In vitro* studies have identified several factors which are likely to modulate Stx release *in vivo*. Hydrogen peroxide produced by neutrophils and contact of STEC with intestinal epithelial cells or macrophages has been shown to enhance Stx2 expression (Wagner *et al*., 2001a; Shimizu *et al*., 2011). In addition, ethanolamine has been identified as an environmental cue which leads to Stx2 induction in the colon (Kendall *et al*., 2012). On the other hand, factors secreted by human microbiota and nitric oxide generated by activated intestinal epithelial cells have been shown to inhibit Stx2 expression and release (Vareille *et al*., 2007; de Sablet *et al*., 2009). Our study using a microaerobic T84 cell infection model shows that low oxygen levels similar to those in the human intestine suppress Stx2 release into the medium. This is in agreement with a recent study which demonstrated lower Stx release by O157 strain EDL933 in simulated ileal and colonic environment media under MA versus AE conditions (Polzin *et al*., 2013). As Stx2 production and release are inherently linked to the phage lytic cycle and therefore bacterial lysis and death (Mühldorfer *et al*., 1996; Wagner *et al*., 2001b) it could be argued that the MA environment in the human gut promotes STEC survival. In addition, *in vivo* experiments in mice have demonstrated that the intestinal epithelium is surrounded by a zone of relative oxygenation resulting from oxygen diffusion from the villous vasculature (Marteyn *et al*., 2010). Although it remains yet to be determined how much oxygen is present at the epithelial surface, it could be speculated that this is sufficient to trigger Stx2 phage lysis and targeted toxin release to the epithelium.

Whereas STEC strains Walla-1 and H1121 express Stx2 only, EDL933 also produces Stx1. In our study, Stx1 expression was also reduced under MA versus AE conditions. However, in contrast to Stx2, Stx1 was detected intracellularly and represented only 2–4% of total Stx released during infection. This is similar to results obtained with STEC cultured in broth or cell culture medium where Stx2 was the main toxin type detected in the supernatant (Strockbine *et al*., 1986; Gobert *et al*., 2007; Shimizu *et al*., 2009). The different localization is likely linked to the different transcriptional regulation of the two Stx types. Whereas Stx2 transcription is mainly driven by the late phage promoter *p*_R_’ and therefore dependent on phage induction and bacterial lysis (Wagner *et al*., 2001b), Stx1 appears to be predominantly expressed from the toxin promoter *p_stx1_* which is independent of the phage lytic cycle so that Stx1 remains localized within the periplasm (Wagner *et al*., 2002; Ritchie *et al*., 2003). Predominant Stx2 release during STEC infection might explain why this toxin type is more closely linked to HUS than Stx1 (Heuvelink *et al*., 1995; Boerlin *et al*., 1999). However, this does not explain why STEC strains expressing both Stx1 and Stx2 appear less virulent than strains expressing Stx2 only in some studies (Pickering *et al*., 1994) which might be linked to interaction between Stx1 and Stx2 phage repressors or competition of the two toxin types for Gb3 binding. In addition, predominant Stx2 release might not always apply in the *in vivo* situation, and preferential Stx1 release in faecal filtrates has been observed in some cases (Cornick *et al*., 2002).

As STEC are non-invasive, released Stxs have to permeate the intestinal epithelial barrier to enter the bloodstream and cause HUS. In contrast to intestinal and renal vasculature, human intestinal epithelium does not appear to express the Stx receptor Gb3 or any other toxin binding site and is resistant to Stx cytotoxicity (Björk *et al*., 1987; Holgersson *et al*., 1991; Schüller *et al*., 2004; 2007[Bibr b52],[Bibr b53]). Several routes of Stx transport across the gut epithelium have been proposed (Schüller, 2011) with Gb3-independent transcytosis being the most investigated mechanism (Acheson *et al*., 1996; Philpott *et al*., 1997; Hurley *et al*., 2001; Malyukova *et al*., 2009). Most of these studies have been performed with purified Stx1 only but experiments with Caco-2 cells have demonstrated that Stx1 and Stx2 are transcytosed by different pathways (Hurley *et al*., 1999). This together with the strong association of Stx2 with HUS emphasizes the need for studies directed at Stx2. In addition, the use of purified Stx instead of STEC infection might neglect the following factors which might be relevant in the *in vivo* situation. (i) Physiological Stx concentrations during STEC infection in the human gut are unknown. Therefore, the use of arbitrary concentrations of purified toxin might lead to artificial results. (ii) A recent study has suggested that partial unfolding of Stx1 during multi-step purification methods may lead to loss of neutrophil binding (Brigotti *et al*., 2011). Therefore, it is possible that changes in Stx conformation could also affect uptake and transcytosis. (iii) Stxs can be released from STEC within outer membrane vesicles (OMVs) (Kolling and Matthews, 1999), and the enclosure within a lipid bilayer may affect the mechanism of uptake and transcytosis. (iv) STEC infection might modify Stx uptake and translocation. In addition to investigating Stx2 translocation during STEC infection, we have also determined the influence of a MA environment similar to that in the human gut on this process. Translocation levels of purified Stx2 in our system were comparable to those published previously (0.02–0.04% after 5 h compared with 0.02% after 4 h respectively) (Hurley *et al*., 2001). In addition, no significant differences were observed between AE and MA conditions indicating that oxygen does not directly affect host cell transcytosis. While similar translocation rates were obtained during AE infection with EDL933, Stx absorption was significantly enhanced during MA infection. Together these results suggest that MA conditions may induce bacterial factors which promote Stx transcytosis. It is tempting to speculate that this could be related to the T3SS and its effector proteins which have been shown to profoundly affect host cell signalling (Croxen and Finlay, 2010). This would be supported by the observation that MA infection with H1121 which does not express an STEC T3SS, does not significantly enhance Stx absorption. In addition, our previous studies have shown enhanced T3S under microaerobiosis (Schüller and Phillips, 2010).

Very few other studies have been conducted to date to investigate the influence of STEC infection on Stx translocation. All studies have been performed under AE conditions and the results are contradictory. While Philpott and colleagues have demonstrated that translocation of purified Stx1 across polarized T84 cells is not altered by simultaneous infection with an Stx-negative STEC laboratory variant (Philpott *et al*., 1997), recent studies have shown stimulation of uptake and/or transcytosis of purified Stx1 by incubation with STEC Stx-deletion mutants or lysates (Lukyanenko *et al*., 2011; In *et al*., 2013). These latter studies have postulated macropinocytosis as mechanism of Stx transport which is supported by formation of actin-rich macropinocytic blebs at apical cell surfaces, localization of Stx in large actin-coated vesicles, and dependence on non-muscle myosin II and Cdc42 (Malyukova *et al*., 2009; Lukyanenko *et al*., 2011; In *et al*., 2013). In contrast, we did not detect any signs of macropinocytosis during STEC infection, and none of the inhibitors used affected Stx2 translocation. Although cytochalasin D and blebbistatin caused a loss of T84 cell barrier function as described previously (Madara *et al*., 1988; Ivanov *et al*., 2005), this did not result in obvious paracellular Stx leakage. It is unlikely that any inhibitory effect of cytochalasin D on Stx transcytosis might be masked by increased paracellular permeability as previous studies have shown maintenance of size restriction in cytochalasin D-treated T84 cells (Madara *et al*., 1988). The discrepancy between our study and those by Kovbasnjuk and colleagues might be due to several factors which include Stx type (Stx2 versus Stx1) and focus on translocation versus uptake which might not be necessarily correlated. In addition, there are differences in experimental protocols. In our study, we have used wild-type STEC instead of co-incubations of arbitrary levels of purified Stx administered together with Stx-negative STEC. Therefore, Stx concentration, conformation as well as degree of association with OMVs are more likely to parallel the *in vivo* situation. In addition, the use of fluorescent tags to label Stx might interfere with biological properties of the conjugate toxins. Furthermore, the morphology of the T84 monolayers appears different in XZ confocal scans which might indicate a different polarization status.

In addition to infection with STEC O157:H7, we have also included an O104:H4 isolate (H1121) from an STEC outbreak in Germany in 2011. This outbreak has been the most severe recorded to date and has affected around 4000 people. The unusually high rate of HUS (22%) suggests increased bacterial virulence (Bielaszewska *et al*., 2011). As mentioned earlier, adherence of H1121 to polarized T84 cells was considerably enhanced compared with the two O157:H7 strains tested. Although microaerobiosis had a similar effect on H1121 compared with O157 strains, amounts of Stx2 released during infection and translocation rates across epithelial monolayers were significantly lower compared with EDL933. Both strains express the same Stx2 subtype (Stx2a). These results suggest that augmented virulence of H1121 is not related to increased Stx expression and transcytosis *in vivo* but is more likely caused by its increased ability to adhere and colonize the human colon. This in turn might lead to enhanced tissue damage due to bacterial factors and the host inflammatory response and thereby promote Stx leakage into the systemic circulation.

In summary, we have demonstrated that a microaerobic environment similar to that in the human gut modulates Stx production, release and translocation during colonic STEC infection. Our observations suggest that low oxygen levels induce STEC factors which promote Stx2 transcytosis across human colonic epithelium. Identification of these factors will remain the challenge for future studies and will lead to a better understanding of Stx absorption into the systemic circulation.

## Experimental procedures

### Cell culture

Human colon carcinoma T84 cells (ATCC CCL-248) were cultured in DMEM/F-12 mixture supplemented with 10% fetal bovine serum (FBS, Sigma) and used between passage 42 and 65. For diffusion chamber experiments, 5 × 10^5^ T84 cells cm^−2^ were seeded on collagen-coated Snapwell filter inserts (12 mm diameter, 0.4 μm pore; Corning Costar). TER was monitored using an EndOhm chamber and EVOM resistance meter (WPI) and values of 1000 to 1500 Ω × cm^2^ after 12–18 days of differentiation indicated establishment of epithelial barrier function. African Green monkey kidney Vero cells (ECACC 84113001) were cultured in DMEM supplemented with 10% FBS. All cells were grown at 37°C in a 5% CO_2_ atmosphere.

### Bacterial strains and culture conditions

STEC prototype strain O157:H7 EDL933 [Stx1a+, Stx2a+; isolate from 1982 outbreak in Michigan/Oregon (Riley *et al*., 1983)] and strain O157:H7 Walla-1 [(Stx2a+; isolate from 1986 outbreak in Walla Walla, Washington state (Ostroff *et al*., 1990)] were provided by Roberto La Ragione (University of Surrey, Guildford). STEC O104:H4 strain H1 1218 0280 (Stx2a+; isolate from the 2011 outbreak in Germany) was obtained from Geraldine Smith (Public Health England, London Colindale). Bacteria were grown standing in LB broth overnight at 37°C. Bacteria were spun down before infection and suspended in serum-free culture medium.

### Infection in a vertical diffusion chamber system

Experiments in VDCs (Harvard Apparatus) were performed as described previously (Schüller and Phillips, 2010) with the following modifications. Polarized T84 cells were infected apically with approximately 10^7^ bacteria. Apical chambers were perfused with air, 5% CO_2_ (aerobic) or 90% N_2_, 5% H_2_, 5% CO_2_ (anaerobic) whereas basal compartments were kept under aerobic conditions. Oxygen concentrations in apical compartments were monitored during the experiment using an ISO2 dissolved oxygen meter (WPI). Chambers perfused with air demonstrated oxygen levels of 18–20% of atmospheric pressure whereas 1.4–1.7% was detected in chambers gassed with anaerobic gas mixture. For some VDC experiments, 100 ng ml^−1^ purified Stx2 (Anne Kane, Tufts Medical Center, Boston, USA), 200 μM NEM, 10 μg ml^−1^ type VI-A HRP, 1 μM cytochalasin D, 50 μM blebbistatin, or 10 μM ML 141 (Sigma) were added to the apical chamber. Incubations were performed for 3–8 h without medium change. At the end of the experiment, apical media were collected and OD_600_ values were determined to assess growth of non-adherent bacteria. Bacteria were pelleted by centrifugation and supernatants and pellets were stored at −20°C until further analysis. Basal media were concentrated with Amicon Ultra centrifugal filter units (Millipore) and stored at −20°C. Membrane filters were cut out from supports, washed twice in cold PBS to remove non-adherent bacteria and processed according to further analysis.

### SEM

T84 cells on filters were fixed with 2.5% glutaraldehyde in 0.1 M phosphate buffer and dehydrated through graded acetone series. Specimens were dried using tetramethylsilane (Sigma), mounted on aluminium stubs, sputter-coated with gold (Polaron SC7640 sputter coater, Quorum Technologies), and viewed with a JEOL JSM 4900 LV or Zeiss Supra 55 VP FEG scanning electron microscope.

### Immunofluorescence staining

T84 cells on filters were fixed in 3.7% formaldehyde in PBS for 10 min and blocked/permeabilized with 0.1% Triton X-100 (Tx-100) and 0.5% BSA in PBS for 20 min. For occludin staining, cells were pre-extracted as recommended by the manufacturer. Cells were subsequently incubated in primary antibodies (polyclonal goat anti-*E. coli* from abcam, polyclonal rabbit anti-occludin from Invitrogen) for 60 min, washed and incubated in Alexa Fluor-conjugated anti-goat or anti-rabbit IgG (Invitrogen) for 30 min. Cell nuclei/bacterial DNA and filamentous actin were labelled with DAPI (Roche) and FITC-conjugated phalloidin (Sigma) respectively. Filters were mounted in Vectashield (Vector Laboratories) and analysed using a fluorescence light microscope (Axio Imager, Zeiss) or confocal laser scanning microscope (LSM 510 Meta, Zeiss).

### SDS-PAGE and Western blot analysis

Bacterial lysates were prepared by suspending bacterial pellets in reducing SDS-PAGE sample buffer. Volumes were adjusted according to bacterial density (OD_600_). For cell lysates, T84 monolayers were lysed in ice-cold RIPA buffer containing 1 mM PMSF, 1 mM Na_3_VO_4_ and protease inhibitor cocktail (1:200, Sigma). Samples were resolved on 12% SDS-polyacrylamide gels and proteins were transferred to PVDF membranes (GE Healthcare). Membranes were blocked with 5% skimmed milk in TBS/0.05% Tween-20 for 60 min and incubated with primary antibodies (mouse anti-Stx1A and anti-Stx2A from the Biodefense and Emerging Infections Research Resources Repository, NIAID, NIH; rabbit anti-GroEL from Sigma; mouse anti-Src and anti-p-Src from Millipore) overnight at 4°C. After washing, blots were incubated with HRP-conjugated goat anti-rabbit or anti-mouse IgG (Sigma) and developed using enhanced chemiluminescence (Immobilon Western, Millipore) and a FluorChem E Imager (ProteinSimple).

### ELISA

Stx in apical supernatants was quantified using a sandwich ELISA as described previously (Acheson *et al*., 1990). Microtitre plates were coated with P1 glycoprotein (Anne Kane, Tufts Medical Center, Boston, USA) and Stx1 and Stx2 were detected using mouse monoclonal antibodies (Biodefense and Emerging Infections Research Resources Repository, NIAID, NIH).

### Vero cell cytotoxicity assay

Apical and concentrated basal supernatants were filter sterilized and 50 μl sample were pipetted into a 96 well culture plate. For neutralization assays, Stx1- or Stx2-specific antibodies were added at a final dilution rate of 1:500 and plates were incubated at 37°C for 90 min to allow Stx-antibody binding. Vero cells were added to each sample (2 × 10^4^ cells in 100 μl) and plates were incubated for 72 h at 37°C in a 5% CO_2_ atmosphere. Cells were washed with PBS, fixed in 3.7% formaldehyde in PBS and stained with 0.1% (w/v) crystal violet in 10% ethanol for 20 min. After elution of the dye in 50% EtOH, crystal violet staining was quantified at OD_595_. When comparing Stx release between different strains or oxygen levels, Stx concentrations in supernatants were adjusted to OD 0.1 or OD 1.0 to account for different bacterial densities in apical media.

### HRP activity assay

Apical and concentrated basal supernatants (20 μl) were incubated with 200 μl HRP reagent (50 mM NaH_2_PO_4_, pH 5.0; 0.1 mg ml^−1^ *o*-dianisidine, 0.003% (w/v) H_2_O_2_) for 20 min in the dark. The reaction was quantified at OD_450_ versus OD_630_.

### Statistics

Statistical analysis was performed using GraphPad Prism 6 software. Student’s paired *t*-test was used to determine differences between two groups. One-way or two-way anova with Tukey’s multiple comparisons test was used for multiple groups. A *P*-value of < 0.05 was considered significant.

## Supporting Information

Fig. S1 Stx2 is the main toxin type released during infection with EDL933. Undiluted apical supernatants (SN ud) or supernatants diluted 1:10 (SN 1:10) from EDL933-infected T84 cells were incubated with anti-Stx1, anti-Stx2 or medium control (no Ab) before Vero cells were added. Vero cell cytotoxicity is evident in SN samples without antibodies and with anti-Stx1 but not with anti-Stx2.

Fig. S2. Correlation of Stx2 concentrations determined by ELISA and VCCA. Polarized T84 cells were infected for 5 h under AE (■) or MA (□) conditions. Stx2 levels in apical supernatants were quantified by sandwich ELISA or VCCA. Data are shown as means ± SEM from three independent experiments performed in duplicate.
