# Supplementary figures and images for: Shiga toxin production and translocation during microaerobic human colonic infection with Shiga toxin-producing E. coli O157:H7 and O104:H4
Source: Cell Microbiol. 2014 Mar 21;16(8):1255–66. doi: 10.1111/cmi.12281 (PMC4231982; doi:10.1111/cmi.12281)

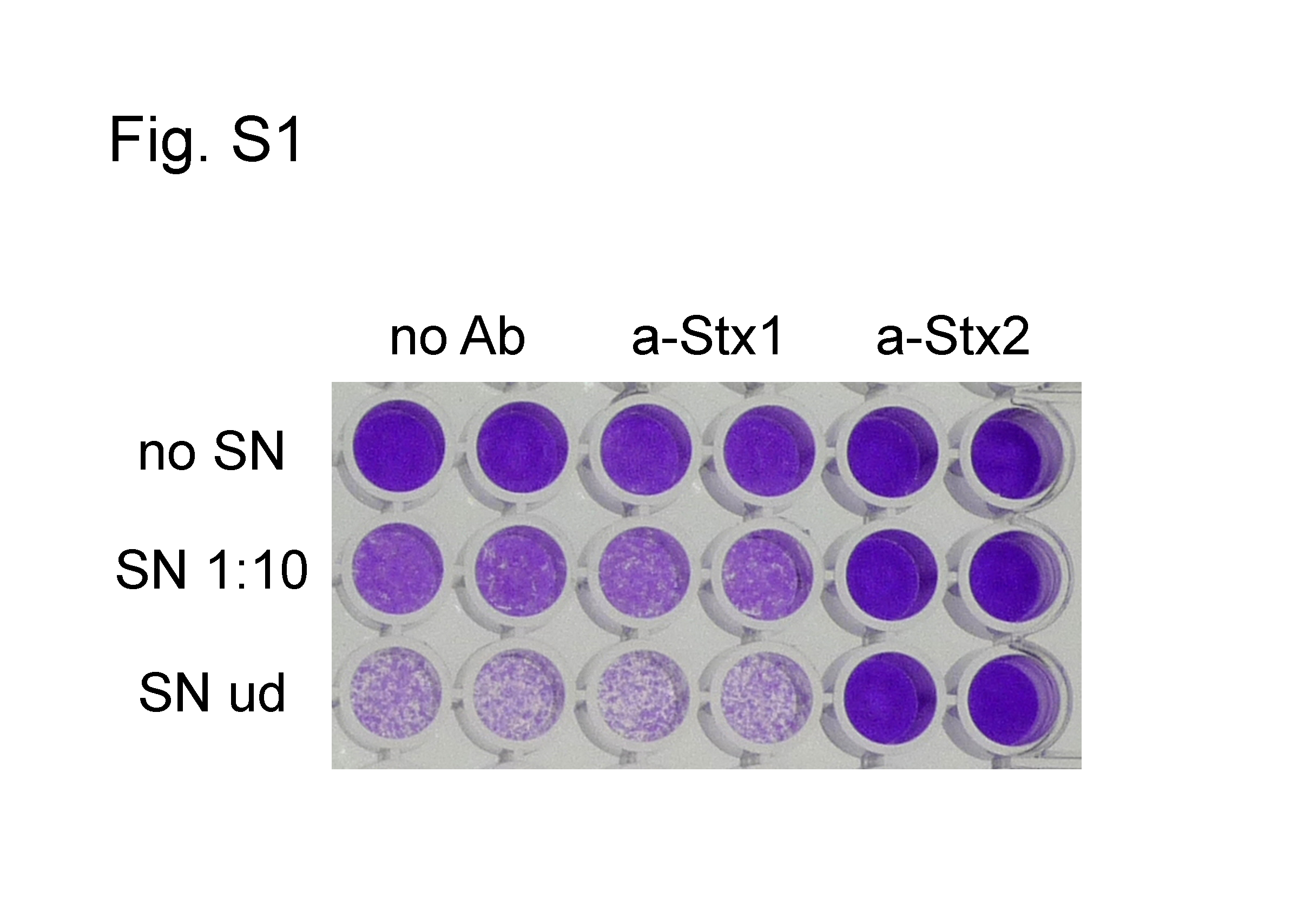

Supplement: Fig. S1 — Stx2 is the main toxin type released during infection with EDL933. Undiluted apical supernatants (SN ud) or supernatants diluted 1:10 (SN 1:10) from EDL933-infected T84 cells were incubated with anti-Stx1, anti-Stx2 or medium control (no Ab) before Vero cells were added. Vero cell cytotoxicity is evident in SN samples without antibodies and with anti-Stx1 but not with anti-Stx2. [file cmi0016-1255-SD1.tif]

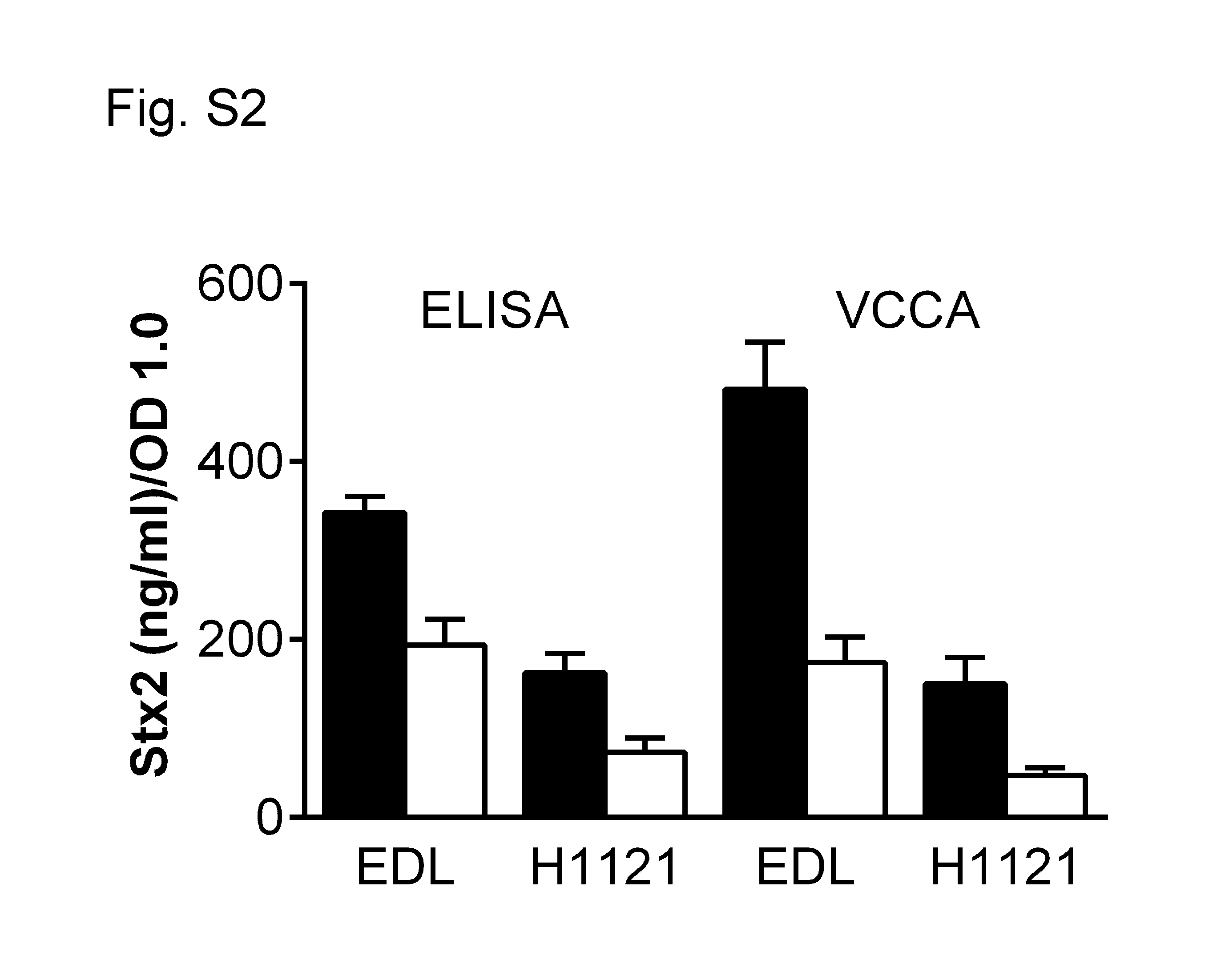

Supplement: Fig. S2. — Correlation of Stx2 concentrations determined by ELISA and VCCA. Polarized T84 cells were infected for 5 h under AE (■) or MA (□) conditions. Stx2 levels in apical supernatants were quantified by sandwich ELISA or VCCA. Data are shown as means ± SEM from three independent experiments performed in duplicate. [file cmi0016-1255-SD2.tif]
